# Quantitative Proteomic Analysis of Castor (*Ricinus communis* L.) Seeds During Early Imbibition Provided Novel Insights into Cold Stress Response

**DOI:** 10.3390/ijms20020355

**Published:** 2019-01-16

**Authors:** Xiaoyu Wang, Min Li, Xuming Liu, Lixue Zhang, Qiong Duan, Jixing Zhang

**Affiliations:** 1 College of Life Science, Inner Mongolia University for Nationalities, Tongliao 028000, China; liuxuminggenes@hotmail.com (X.L.); 15334940513@163.com (L.Z.); duanqiong0209@163.com (Q.D.); 2 Inner Mongolia Key Laboratory for Castor, Tongliao 028000, China; 3 Inner Mongolia Industrial Engineering Research Center of Universities for Castor, Tongliao 028000, China; 4 Inner Mongolia Collaborate Innovation Cultivate Center for Castor, Tongliao 028000, China; 5 Horqin Plant Stress Biology Research Institute of Inner Mongolia University for Nationalities, Tongliao 028000, China; 6 College of Agriculture, Inner Mongolia University for Nationalities, Tongliao 028000, China; lm@imun.edu.cn

**Keywords:** *Ricinus communis* L., cold stress, seed imbibition, iTRAQ, proteomics

## Abstract

Early planting is one of the strategies used to increase grain yield in temperate regions. However, poor cold tolerance in castor inhibits seed germination, resulting in lower seedling emergence and biomass. Here, the elite castor variety Tongbi 5 was used to identify the differential abundance protein species (DAPS) between cold stress (4 °C) and control conditions (30 °C) imbibed seeds. As a result, 127 DAPS were identified according to isobaric tag for relative and absolute quantification (iTRAQ) strategy. These DAPS were mainly involved in carbohydrate and energy metabolism, translation and posttranslational modification, stress response, lipid transport and metabolism, and signal transduction. Enzyme-linked immunosorbent assays (ELISA) demonstrated that the quantitative proteomics data collected here were reliable. This study provided some invaluable insights into the cold stress responses of early imbibed castor seeds: (1) up-accumulation of all DAPS involved in translation might confer cold tolerance by promoting protein synthesis; (2) stress-related proteins probably protect the cell against damage caused by cold stress; (3) up-accumulation of key DAPS associated with fatty acid biosynthesis might facilitate resistance or adaptation of imbibed castor seeds to cold stress by the increased content of unsaturated fatty acid (UFA). The data has been deposited to the ProteomeXchange with identifier PXD010043.

## 1. Introduction

Cold stress is one of the major threats to plant growth, spatial distribution, agricultural productivity, and crop yield [1]. Most temperate plants, such as winter wheat, oats, and barley, can acquire cold acclimation and tolerate ice formation in their tissues; however, many important crops, such as rice, maize, and soybeans, are sensitive to cold stress and incapable of cold acclimation [2]. *Ricinus communis* L. (Euphorbiaceae) is an important non-edible oilseed crop originating in tropical regions but cultivated in many subtropical regions worldwide. The seed oil of castor bean is mainly used for pharmaceutical and industrial applications, as it is a rich source of ricinoleic acid, an unusual hydroxylated fatty acid [3]. Castor beans can be cultivated in some unfavorable environments, such as saline and drought conditions, where other crops would not grow and produce a good yield [4]. However, castor bean is sensitive to cold stress in temperate regions, where the temperature drops frequently during the early growing season. It has been reported that temperature below 20 °C can dramatically decrease seed germination capability [5]. Thus, a prime target for breeding efforts is to improve seed germination under cold stress. A combination of traditional and molecular assistant selection breeding is an effective strategy for generating stress-tolerant and widely adapted castor varieties. Therefore, it is imperative to understand the molecular response to cold stress and to identify some novel responsive genes or proteins in castor bean with strong potential for the improvement of cold tolerance by genetic engineering.

Seed germination, the first and important phase for plant propagation, starts by seed imbibition, leading to embryo transition from a state of quiescence in a dry seed to a state of highly active metabolism, and terminates with embryonic axis elongation [6]. Generally, seed germination can be divided into three phases: a rapid phase of water uptake (Phase I), followed by a plateau phase of water uptake (Phase II), in which grain morphology and structure obviously change and the radical and germ appear, and post-germination (Phase III), a rapid phase of water uptake with the initiation of growth [7,8]. During seed imbibition, phase I represents the initial seed germination stage, in which necessary structures and enzymes are present and storage substances, such as starch, proteins, and lipids, which provide energy and nutrition for seed germination, begin to be activated [8].

A handful of studies have proved that the transcript level of gene expression does not necessarily directly correlate well with abundance of corresponding protein species [9,10]. Since proteins were a direct effector of plant stress response, it was highly important to investigate changes in proteome level to identify potential protein markers whose abundance changes could be linked with changes in physiological indices under cold stress [11]. Early study showed that the abundance of cold-regulated/late embryogenesis-abundant (COR/LEA) proteins were enhanced by cold stress [12]. Several reactive oxygen species scavenging enzymes involved in metabolism of ascorbate-glutathione cycle were up-accumulated under cold stress [13]. Up-accumulation of chaperones, especially various heat shock proteins (HSPs), can play crucial roles in preventing protein misfolding caused by low temperature [13]. Recently, high-throughput proteomic technology was widely used to identify a set of differential abundance protein species (DAPS) associated with cellular responses to cold stress in diverse plants [1,14]. For example, 173 DAPS associated with carbohydrate and energy metabolism, translation and posttranslation modification, stress response, signal transduction etc. were detected in maize leaves after cold stress [1]. These studies can significantly contribute to our understanding of cold response. However, less proteome analyses were applied to elucidate the molecular mechanism of cold adaptation or resistance in seed germination compared to seedlings.

To date, several proteomics analyses identified a range of proteins related to germinating or developing castor seeds [15,16,17,18,19,20]. Several classes of seed reserve proteins such as 2S albumins, legumin-like and seed storage proteins, and proteins involved in plant defenses against biotic and abiotic stresses, were identified from developing castor seeds using two-dimensional electrophoresis (2-DE) [18]. Some important proteins involved in fatty acid metabolism, seed storage proteins, toxins, and allergens associated with developing castor oil seeds were identified by employing isotope coded protein label (ICPL) and isobaric tag for relative and absolute quantification (iTRAQ) technologies [20]. Fourteen proteins were identified in the endoplasmic reticulum of germinating castor seeds; ten of these proteins were concerned with roles in protein processing and storage, and lipid metabolism [15]. However, the proteomics analyses of early seed imbibition for castor under cold stress are rare. Recently, iTRAQ is more accurate and reliable for quantitation of protein species than traditional 2-DE analysis and is currently widely used for identifying cold-accumulated protein species in many plants [1,21].

In this study, we used iTRAQ-based proteomics to detect DAPS between cold-stressed imbibed seeds and unstressed control seeds. Possible biological functions and potential effects of these DAPS on cold tolerance were discussed. This analysis revealed complex changes at the proteomics level in early imbibed castor seeds under cold stress and provided new information concerning the plant responses to cold stress.

## 2. Results

### 2.1. Germination Analysis of Castor Beans During Imbibition

**Figure 1:**
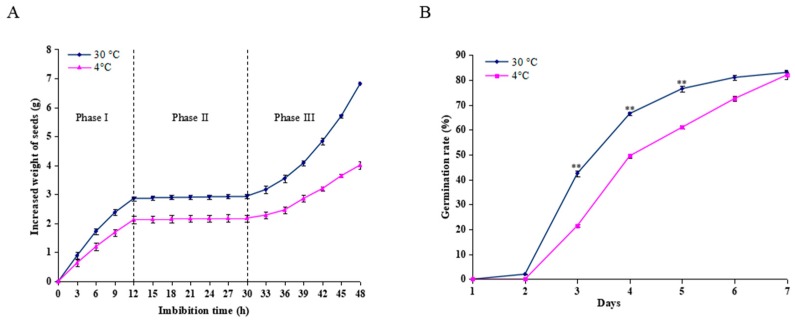
Effect of cold stress on seed imbibition and germination. (**A**) Triphasic pattern of water uptake under the cold stress (4 °C) and control conditions (30 °C). (**B)** Seed germination rate was calculated after the seeds imbibed at 4 °C and 30 °C for 12 h. For the cold stress treatment, the seeds were imbibed at 4 °C for 12 h, and then transferred to 30 °C for germination, and for the control, the seeds were allowed to germinate at a constant 30 °C only. Values represent the means ± SD from three fully independent biological replicates. * *p* < 0.05, ** *p* < 0.01 by Student’s *t*-test.

The changes in water uptake were assessed by measuring changes in the seed weight during imbibition at 30 °C and 4 °C. As shown in Figure 1A, seeds imbibed at 30 °C and 4 °C revealed a triphasic pattern during germination; however, the seeds imbibed at 30 °C could absorb more water than those at 4°C during the same period. Water uptake increased rapidly before 12 h (Phase I), followed by a plateau of seed imbibition from 12-30 h (Phase II) and rapid water uptake after 30 h (Phase III). The seed radical began to emerge during Phase II. However, in the seeds imbibed at 4 °C for 12 h, and then transferred to 30 °C, germination was retarded by 1 day compared to the control conditions. Only 21.5% of seeds germinated at day 3, in contrast to the imbibed seeds at a constant 30 °C, with 42.5% (Figure 1B). Thus, the imbibed seeds in the Phase I (12 h) were collected for further proteomic analysis.

### 2.2. Primary Data Analysis and Protein Identification

iTRAQ-based comparative proteome was used to identify the DAPS between cold stress (4 °C) and control conditions (30 °C) imbibed seeds. IPeak identified a total of 38863 spectra, 8280 peptides and 1670 proteins. In total, 74% proteins included at least two unique peptides. The mass of the identified proteins with 0–50, 51–100, and >100 kDa accounted for 64.4, 30.2, and 5.4% separately. 26 low molecular weight proteins (Mr > 10 kDa) and 91 high molecular weight proteins (Mr > 91 kDa) were identified using the iTRAQ strategy. The distribution of protein sequence coverage with 40–100, 30–40, 20–30, 10–20, and under 10% variations accounted for 8.7, 7.7, 13.5, 23.9 and 46.2%, respectively (Table S2). The mass spectrometry proteomic data of the present study have been deposited to the ProteomeXchange with identifier PXD010043 (http://proteomecentral.proteomexchange.org/cgi/GetDataset?ID=PXD010043).

### 2.3. Identification of DAPS by iTRAQ

A protein species was considered differentially accumulated when it exhibited a fold change >1.2 and *p* value <0.05. Based on these criteria, 127 DAPS were identified, of which, 109 were up-accumulated and 18 were down-accumulated under cold stress versus control conditions. Detailed information is provided in Table S3.

### 2.4. Bioinformatics Analysis of DAPS Identified by iTRAQ

**Figure 2:**
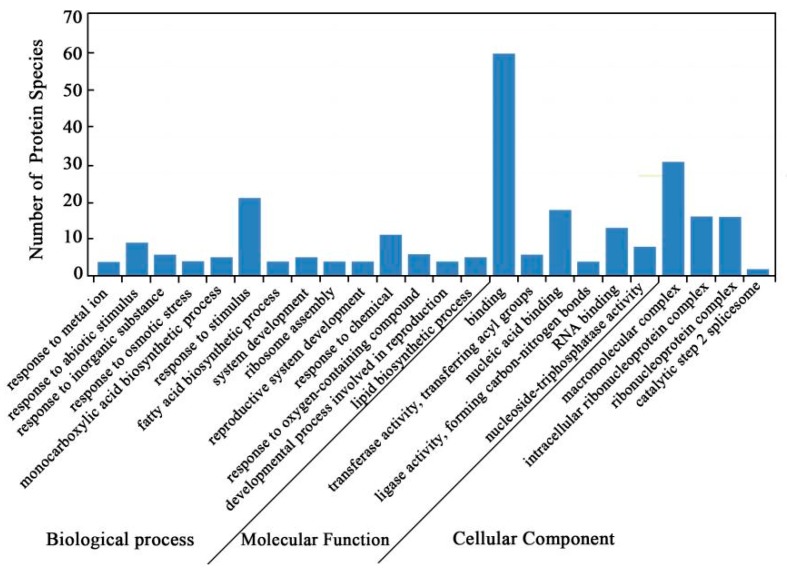
GO annotation of DAPS identified by the iTRAQ.

Gene ontology (GO) annotations were carried out to identify the significantly enriched functional groups of DAPS. A total of 84 DAPS under cold stress versus control conditions were classified into 24 functional groups (Figure 2, Tables S1–S4), of which biological processes accounted for 14 GO terms (the most representative were “response to stimulus”), cellular components accounted for 4 GO terms (the most representative were “macromolecular complex”), and molecular functions accounted for 6 GO terms (the most representative were “binding”).

**Figure 3:**
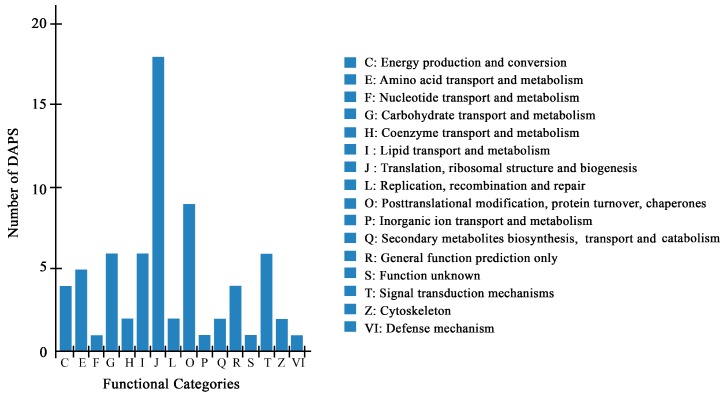
COG classification of DAPS identified by the iTRAQ.

A total of 70 DAPS by iTRAQ were classified into 16 categories of Clusters of Orthologous Groups of proteins (COG), among which, “translation, ribosomal structure and biogenesis” represented the largest group (group J, 18 DAPS), followed by “posttranscriptional modification, protein turnover, chaperones” (group O, 9 DAPS) (Figure 3, Tables S2–S4).

To further explore the biological functions of these proteins, 56 DAPS were mapped to 16 pathways in the KEGG database (Tables S3 and S4). These annotated protein species were significantly enriched in the following pathways: “fatty acid biosynthesis”, “biotin metabolism”, “fatty acid metabolism”, “cyanoamino acid metabolism”, and “ribosome” (Table 1).

### 2.5. Confirmation of DAPS by ELISA

**Figure 4:**
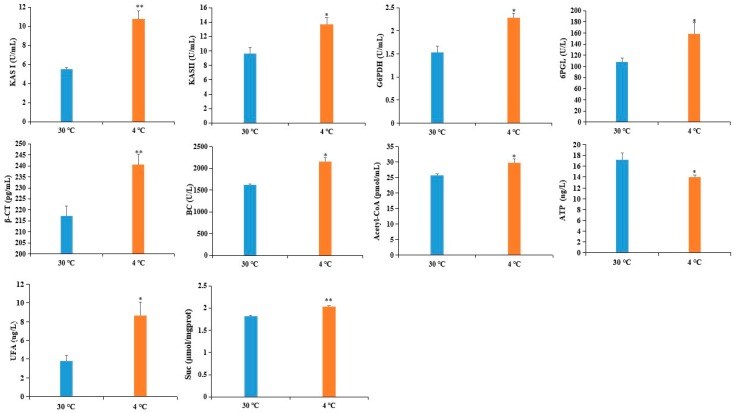
Validation of DAPS by ELISA. Values represent the means ±SD from three fully independent biological replicates. * *p* < 0.05, ** *p* < 0.01 by Student’s *t*-test.

To validate the reliability of the DAPS as determined by iTRAQ, we used ELISA to assess the expression level of six DAPS. Four of the six DAPS were involved in fatty acid metabolism, including β-ketoacyl-acyl carrier protein synthase (KAS) II (KASII), KASI, biotin carboxylase (BC) subunit of Het-ACCase, and β-carboxyltransferase (β-CT) subunit of Het-ACCase, whereas the other two DAPS were involved in the pentose phosphate pathway: 6-phosphogluconolactonase (6PGL) and glucose-6-phosphate 1-dehydrogenase (G6PDH). As shown in Figure 4, the level of six DAPS was increased by cold stress, which showed a good correlation between the ELISA and iTRAQ-based datasets.

### 2.6. Transcriptional Analyses of the Corresponding Genes Encoding DAPS

To know the correlation between the abundance of DAPS and the transcript level of their corresponding genes, eight up-accumulated DAPS were selected for qRT-PCR analyses. The eight DAPS were dehydrin Xero (B9S696), glutathione peroxidase (GPX) (B9RCA6), late embryogenesis abundant protein D-34 (LEA D-34) (B9RTR0), LEA D-34 (B9S3Z7), SNF1-related protein kniase (SnRK1) α catalytic subunit (B9SVJ9), KAS II (Q41134), eukaryotic translation initiation factor 5A (IF5A) (B9STQ5), protein phosphatase 2c (PP2C) (B9SB19). The results showed that the eight selected DAPS can be clustered into two groups, i.e., group I, up-regulated at both transcript and protein level; Group II, no change at transcript level while up-accumulated at protein level (Table 2). This discrepancy between the mRNA and protein expression profiles indicated that the abundance of protein depends not only on the transcript level but also on transcript stability, post-transcriptional regulation, post-translational modifications, and protein degradation [9,10].

## 3. Discussion

Castor beans, which possess a high economic value, are very sensitive to cold stress, especially at the germination stage. Transcriptome analysis has identified many differentially expressed genes (DEGs) mainly related to plant secondary metabolism in germinated seeds under cold stress [22]. However, the mechanisms underlying the effects of cold stress on early seed imbibition are largely unknown. This initial proteomics analysis of castor seeds during early imbibition identified several cold-accumulated protein species and unraveled a complex cellular network affected by cold stress. In this study, 127 DAPS were identified, among which, 109 were up-accumulated and 18 were down-accumulated under cold stress compared to control conditions. Unsurprisingly, these DAPS included some well-known stress-inducible proteins, such as the LEA, dehydrin. In addition, some cold-accumulated protein (methionine aminopeptidase) identified here have been verified in other plants based on iTRAQ strategy. Furthermore, this approach also identified some novel proteins that were not previously known to be associated with cold stress response. It has been reported that mature dry seeds can rapidly restart metabolic activity including protein synthesis after imbibing water. De novo protein synthesis was necessary for seed germination in rice [23]. Our results showed that “ribosome” was the significantly enriched pathway involved in early seed imbibition under cold stress. Dry seed also contained a myriad of “long-lived mRNA”, which was thought to be translated after imbibition [23]. It is interesting to further investigate if de novo transcription was required for germination of castor seeds by control-treated imbibed seeds versus dry seeds and translation of long-lived mRNAs was induced or regulated by cold stress during the germination of castor seeds. In brief, bioinformatics analysis revealed that 84 and 70 DAPS were annotated in 24 GO functional groups and 16 COG categories, respectively. In total, 56 DAPS were mapped into 16 KEGG pathways. The possible biological significance of some key DAPS and their relevant metabolic pathways in cold stress adaptation are discussed below.

### 3.1. DAPS Involved in Translation and Posttranslational Modification

Fifteen DAPS, including eleven ribosomal proteins (RPs) and four elongation factors, were up-accumulated in cold-treated imbibed seeds (Table 3, Table S3). RPs are essential for protein synthesis and play a critical role in metabolism, cell division and growth, and regulation of cold stress [21,24]. For example, three soybean ribosomal protein genes were induced by low temperature treatment [25]. Recently, there is increasing evidence that shows direct links between RPs and cold stress. Wang et al. reported that all the DAPS involved in mature ribosome assembly and translation processes were increased in maize leaves after a 12 h cold treatment [1]. Thus, up-accumulation of RPs in cold-treated imbibed castor seeds might be required for de novo transcription or participate as regulatory components in response to cold stress. Elongation factor Tu (EF-Tu), which is responsible for the elongation phase of protein synthesis, has been extensively studied in plant responses to various environmental challenges, such as cold and heat stresses [26]. In the present study, the abundance of EF-Tu was up-accumulated under cold stress, which agrees with the previous study that cold stress can increase the protein level of EF-Tu in rice [27,28]. The eukaryotic translation initiation factor 5A (eIF5A) promotes the first peptide bond formation at the onset of protein synthesis [29]. Plant eIF5A is involved in multiple biological processes, including protein synthesis regulation, translation elongations, mRNA turnover and decay, and abiotic stress responses [30,31,32]. For example, Wang et al. reported that transgenic yeast and poplar expressing TaeIF5A displayed elevated protein levels combined with improved abiotic stresses tolerance [33]. Elongation factor 1α promotes codon-directed binding of aminoacyl-tRNA (aa-tRNA) in the ribosome [34]. Transgenic plants with transgene AtEF1α were more tolerant to NaCl than the wild-type [35]. The increased accumulation of four elongation factors might confer cold tolerance by promoting protein synthesis or regulating physiological pathways. N-terminal Met excision (NME) is a process by which methionine aminopeptidase (MAP) specifically removes the first Met in most newly synthesized proteins. Overexpression of barley DNA-binding MAP in Arabidopsis exhibited stronger freezing tolerance compared to the wild type [36]. The abundance of MAP observed in this study showed increased accumulation in cold-treated imbibed seeds, which agrees with the recent report that cold stress increased the MAP level in petunia [21]. These results led us to speculate that the responsiveness of MAP to cold stress might be a common event that deserves further investigation.

Posttranslational modifications play critical roles in the regulation of abiotic stresses, such as ubiquitination and phosphorylation [37]. The ubiquitination-proteasomal pathway has been implicated in diverse aspects of eukaryotic cellular regulation due to its ability to degrade intracellular protein [38,39]. Ubiquitin-activating enzymes catalyze the first step in the ubiquitination reaction, which activates ubiquitin and transfers the activated Ub to a ubiquitin-conjugating enzyme to form an E2-Ub thiolester [40]. The proteasome plays a fundamental role in retaining cellular homeostasis and is the major cellular proteolytic machinery responsible for the degradation of both normal and damaged proteins [41]. The increased accumulation of three protein species involved in the ubiquitin/26S proteasome system might contribute to potentially harmful polypeptide degradation (Table S3). Heat shock proteins (HSPs) are highly conserved proteins that are present in organisms, function as “molecular chaperones”, promote the degradation of abnormal proteins and prevent the aggregation of denatured proteins [42,43]. Our results showed that the abundance of three heat-shock proteins was increased under cold stress (Table S3).

### 3.2. DAPS Involved in Stress Response

Cold stress can result in overproduction of reactive oxygen species (ROS). ROS can perturb cellular redox homeostasis and lead to oxidative damage to membrane lipids, nucleic acids, and proteins [21]. To relieve cellular damage by ROS, plants have developed ROS scavenging systems including antioxidants and antioxidant enzymes [44]. GPX is a ubiquitous enzyme in plant cells that use glutathione to reduce H_2_O_2_ and lipid hydroperoxides [45,46]. Thus, it was not surprising that the abundance of GPX was increased in cold-stressed imbibed seeds (Table S3). It has been reported that overexpression of GPX can enhance the growth of transgenic tobacco under cold and salt stresses [47]. LEA proteins are involved in many physiological processes, act as protectors of enzyme activities and stabilize membranes associated with anionic phospholipid vesicles at freezing temperature [48,49]. Consistent with previous findings, we identified four LEA proteins that showed increased accumulation in cold-treated lines compared to control lines (Table S3). Dehydrins, which are known as group 2 or D-11 family LEA proteins, can protect the cell against damage caused by stress. A high level of dehydrin transcripts or proteins is closely associated with cold tolerance in numerous plants such as Arabidopsis, rice and Rhododendron [50,51,52]. The increased accumulation of dehydrin may facilitate the resistance or adaptation of castor to cold stress (Table S3). Early responsive to dehydration (ERD) genes can be rapidly induced to counteract abiotic stresses, such as drought, low temperature or high salinity. Overexpression of VaERD15 in Arabidopsis resulted in higher cold tolerance and accumulation of antioxidants compared to wild type under cold stress [53]. The increased abundance of ERD was observed in cold-treated imbibed seeds (Table S3).

### 3.3. DAPS Involved in Carbohydrate and Energy Metabolism

Carbon metabolism provides the necessary energy for subsequent plant growth and development during germination. Simultaneously, carbon metabolism could be an effective connection with other metabolic processes [54]. The pentose phosphate pathway (PPP) is a central metabolic pathway including the irreversible oxidative pathway and reversible non-oxidative pathway, which is catalyzed by several different enzymes such as G6PDH, 6PGL and ribulose-phosphate 3-epimerase (RPE) [55,56,57]. G6PDH catalyzes the first and rate-limiting enzyme of PPP by converting glucose-6-phosphate to 6-phosphogluconolactone. 6PGL catalyzes the hydrolysis of 6-phosphogluconolactone, which was thought to occur spontaneously [57]. Numerous studies regarding G6PDH function in response to abiotic stresses have been performed. Overexpressing of PsG6PDH in transgenic tobacco resulted in enhanced cold tolerance [58]. In this study, the increased accumulation of G6PDH, 6PGL and RPE might provide more reducing equivalent NADPH for anabolic pathways including fatty acid synthesis, and carbon skeletons for the synthesis of acetyl-CoA, etc. (Table S3).

### 3.4. DAPS Involved in Lipid Transport and Metabolism

Fatty acids (FAs) are major components of cell or organelle membrane lipids. FAs are also precursors of messenger compounds such as jasmonic acid and phosphatidylinositol, which play key roles in certain signal transduction pathways, and are used as substrates for the synthesis of storage lipids that are important materials for seed germination and provide energy for humans [59,60,61,62,63]. During FA biosynthesis, acetyl-CoA carboxylase (ACCase) catalyzes the committed step of the de novo FA biosynthesis pathways by converting acetyl-CoA to malonyl-CoA. KAS is vital for carbon chain condensation and elongation from C4-C18. KASI has high activity when butyryl- to myristyl-ACP (C4:0-C14:0 ACP) is used as the substrate to produce hexanoyl- to palmitoyl-ACP (C6:0-C16:0 ACP), whereas KASII catalyzes the last condensation reaction of palmitoyl-ACPs to stearoyl-ACPs [64]. It is well known that alteration of the lipid composition of cell membrane is associated with cold tolerance [65]. The increased accumulation of four DAPS related to FA biosynthesis might adapt castor to cold stress by preventing membrane transition from liquid crystalline phase to gel phase (Table 4). This inference was also supported by our observation that the content of UFA was obviously increased in cold-treated imbibed seeds (Figure 4).

### 3.5. DAPS Involved in Signal Transduction

SnRK1 complex is a heterotrimeric complex composed of a α catalytic subunit that interacts with two other subunits [66]. SnRK1 α catalytic subunit triggers vast transcriptional and metabolic reprogramming and promotes tolerance to adverse conditions [67]. Several evidences have suggested that SnRK1 had the potential to regulate the carbohydrate metabolism of higher plants. For instance, antisense expression of the SnRK1 α catalytic subunit resulted in the reduction of sucrose synthase gene expression in leaves and tubers [68]. Our study showed that the increased level of SnRK1 α catalytic subunit might regulate sucrose metabolism. This inference was supported by our observation that the content of sucrose was increased in cold-treated imbibed seeds (Figure 4). Rosnoblet et al. showed that the amount of SnRK1 gamma subunit in the radical decreased during imbibition and was no longer detectable in the protruded radical [69]. However, conditions that block germination of imbibed seeds, including low water potential or ABA, maintain SnRK1 gamma subnuit expression [70]. Consistent with previous findings, up-accumulation of SnRK1 gamma subunit was observed in cold-treated imbibed seeds (Table S3). Reversible protein phosphorylation mediated by protein kinases and phosphatases is a central mechanism for modulating a body of cellular processes such as signaling transduction, cell division and development [71,72]. Serine/threonine protein phosphatases 2C (PP2C) have been suggested to play an important role in stress signaling [73]. Overexpression of ZmPP2C2 in tobacco enhanced cold tolerance [74]. Consistent with these findings, we identified two PP2Cs which was up-accumulated 1.21- and 1.42- fold in cold-treated lines compared to the control lines (Table S3). Purple acid phosphatase (PAP) represents a diverse group of acid phosphatases in animals, microorganisms, and plants that catalyze the hydrolysis of phosphate esters and anhydrides. It has been reported that the expression of the *GmPAP3* gene can be induced by abiotic stresses, such as salinity and drought. The GmPAP3 transgenic Arabidopsis displayed higher abiotic stress tolerance, which was probably related to alleviation of oxidative damage [75]. Our results showed that the abundance of purple acid phosphatase was increased in cold-treated imbibed seeds (Table S3).

### 3.6. A Proposed Metabolic Pathway for Ricinus Communis During Early Seed Imbibition in Response to Cold Stress 

**Figure 5:**
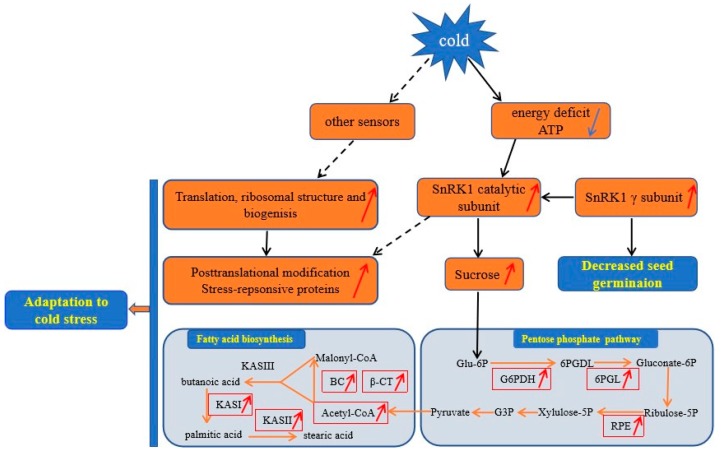
A proposed model for *Ricinus communis* during early seed imbibition in response to cold stress. The up-accumulated DAPS and increased content of acetyl-CoA and sucrose were indicated by the red arrow; the decreased content of ATP were indicated by blue arrow. Abbreviations: Glu-6P, Glucose 6-phosphate; 6PGDL, 6-phosphogluconolactone; G6PDH, glucose-6-phosphate 1-dehydrogenase; 6PGL, 6-phosphogluconolactonase; Gluconate-6P, 6-phosphogluconate; Ribulose-5P, Ribulose-5-phosphate; Xylulose-5P, Xylulose-5-phosphate; RPE, Ribulose-phosphate 3-epimerase; G3P, Glyceraldehyde 3-phosphate; KASI, β-ketoacyl-acyl carrier protein synthase I; KASII, β-ketoacyl-acyl carrier protein synthase II; KASIII, β-ketoacyl-acyl carrier protein synthase III. Solid lines denoted proven connections in plants, whereas broken lines represented connections that might exist in plants.

Based on the above findings and the data presented here, we proposed a view of resistance and adaptation strategies of early seed imbibition for castor under cold stress (Figure 5). Cold stress is partly decoded as an energy-deficiency signal, regardless of their site and mode of perception [76]. SnRK1 α subunit in plants can sense the energy deficit to trigger extensive transcriptional reprogramming that contribute to restoring homeostasis and elaborating long-term responses for adaptation [67]. In our study, cold stress resulted in energy deprivation (Figure 4). Upon sensing the energy deficit, up-accumulation of the SnRK1 α subunit in cold-treated imbibed seeds regulated the sucrose metabolism to produce more substrate for PPP. PPP is an effective connection with other metabolic processes. The up-accumulation of rate-limiting enzymes (G6PDH and 6PGL) and RPE involved in PPP provided more acetyl-CoA for fatty acid synthesis (Figure 4). Chilling stress can impair membrane permeability by the transition of membrane lipids from liquid-crystalline phase to gel phase [77]. Tolerance to chilling stress is closely connected with the fatty acid desaturation of plant membrane lipids [78]. In this study, up-accumulation of some important DAPS significantly enriched in fatty acid biosynthesis might facilitate the resistance or adaptation of imbibed castor to cold stress by increasing the content of UFA (Figure 4).

The absence of a SnRK1 gamma subunit (LeSNF4) in tomato contribute to the transition to the “germination/growth” mode rather than the maintenance of “maturation/dormancy” metabolic state. However, conditions that block germination of imbibed seeds, including ABA and FR light, can maintain LeSNF4 expression [70]. Therefore, the up-accumulation of SnRK1 gamma subunit in cold-imbibed seeds might be one of the reasons for the decreased seed germination ability. Energy deficiency is sensed by the SnRK1 that trigger the repression of genes involved in protein synthesis [79]. Up-accumulation of all mature ribosome assembly and translation in cold-imbibed seeds might be regulated by other sensors to confer cold tolerance by producing more important proteins, such as cold-responsive proteins. Chilling stress can cause dysfunction/denaturation of structural and functional protein. Some DAPS involved in the ubiquitin/26S proteasome system might be responsible for maintaining functional protein conformations and contributing to potentially harmful polypeptides degradation.

## 4. Materials and Methods 

### 4.1. Plant Materials and Stress Treatment

The *ricinus communis* seeds (genotype Tongbi 5) used in this work were kindly supplied by the Tongliao Academy of Agricultural Sciences, China. Castor seeds were sown on 15 April and harvested on 1 October, 2017 in experimental field of Inner Mongolia University for Nationalities, China. This genotype was the elite variety with high seed yield potential (average up to 2316.03 kg/hm^2^) and widely cultivated in China [80]. Briefly, 50 sterilized seeds of each biological replicate with similar size and weight were sown in Petri dishes (d = 12 cm) with filter papers soaked in 16 mL sterile distilled water at 30 °C or 4 °C for 7 days. The weight of the imbibed seeds was recorded every 3 h to calculate the water content of the seeds. The seeds were allowed to germinate at a constant 30 °C for optimal germination and at 4 °C for 12 h and then transferred to 30 °C for cold germination. Three biological replicates were conducted. Germination was determined when the tip of radical grew free of the seed coat [81].

### 4.2. Protein Extraction

Four Petri dishes of 50 sterilized seeds each (a total of 200 seeds) as one biological replicate were imbibed under 4 °C for 12 h along with the seeds imbibed at 30 °C for 12 h as the control. Two biological replicates were conducted for iTRAQ-based comparative proteomics analysis. Cold- and control-treated imbibed seeds were ground into powder with liquid nitrogen. The powder was slowly stirred into a beaker with 100 mL preboiling 2% (*v*/*v*) sodium dodecyl sulfate (SDS) solution and 2 mM phenylmethylsulfonyl fluoride (PMSF) (final concentration) for 3 min. The suspension was rapidly cooled, transferred to two new 50 mL tubes, sonicated for 30 min on ice and then centrifuged at 20,000× *g* for 15 min. The upper oil layer and lower precipitate were discarded, and the middle clear protein solution was dried by lyophilization and concentrated to 5 mL. The protein solution was mixed well with 30 mL 10% chilled trichloroacetic acid (TCA) acetone and incubated at −20 °C overnight. After centrifugation at 4 °C and 20,000× *g*, the supernatant was discarded. The precipitate was washed three times with chilled acetone. The pellet was air-dried and dissolved in lysis buffer (7 M urea, 2 M thiourea, 4% NP40, and 20 mM Tris-HCl, pH 8.0–8.5). The suspension was sonicated at 200 W for 5 min and centrifuged at 4 °C at 30,000× *g* for 15 min. The supernatant was then collected. The sample protein concentration was determined with the Bradford assay using BSA as the calibrant. The quality of the protein sample was measured by SDS-PAGE.

### 4.3. Protein Digestion and iTRAQ Labeling

A total of 100 µg protein for each sample was digested with Trypsin Gold (Promega, Madison, WI, USA) with the ratio of protein: trypsin = 20:1 at 37 °C for 16 h. Peptide samples were labeled with 8-plex iTRAQ reagents (Applied Biosystems, Foster City, CA, USA) according to the manufacturer’s protocol. The control replicates (30 °C) were labeled with the iTRAQ tags 114 and 116, and the cold-treated sample replicates were labeled with the iTRAQ tags 117 and 121. The labeled peptide mixtures were pooled, dried by vacuum centrifugation and fractionated by strong cationic exchange (SCX) chromatography.

### 4.5. Fractionation by SCX

The Shimadzu LC-20AB HPLC Pump system (Kyoto, Japan) was used for SCX chromatography. The iTRAQ-labeled peptide mixtures were reconstituted with 4 mL buffer A (25 mM NaH_2_PO_4_ in 25% acetonitrile, pH 2.7) and loaded onto a 4.6×250 mm Ultremex SCX column containing 5 µm particles (Phenomenex). The peptides were eluted with a gradient of buffer A for 10 min, buffer B (25 mM NaH_2_PO_4_, 1 M KCl in 25% acetonitrile, pH 2.7) for 25 min, and 35–80% buffer B for 1 min. Elution was monitored by measuring absorbance at 214 nm, and fractions were collected every minute. The eluted peptides were pooled as 15 fractions, desalted with a Strata X C18 column (Phenomenex) and vacuum-dried.

### 4.6. LC-ESI-MS/MS Analysis by Q-Exactive

Each fraction was re-dissolved in buffer A (5% acetonitrile, 0.1% formic acid) and centrifuged at 20,000× *g* for 10 min; the final concentration of peptides was approximately 0.5 µg/µL. Then, 8 µL supernatant was loaded on a LC-20AD nanoHPLC (Shimadzu, Kyoto, Japan) by the autosampler onto a C18 trap column, and the peptides were eluted onto an analytical C18 column (inner diameter, 75 µm) packed in-house. The samples were loaded at 8 µL/min for 4 min, and then the 40-min gradient was run at 300 nL/min starting from 2–35% buffer B (95% acetonitrile, 0.1% formic acid), followed by 5 min linear gradient to 80%, maintained at 80% buffer B for 4 min, and finally returned to 5% in 1 min.

Data acquisition was performed in the Q-Exactive (ThermoFinnigan, San Jose, CA, USA) mass spectrometer with a mass/charge (*m*/*z*) scanned range of 350–2000 Da. Intact peptides and ion fragments were detected in the Orbitrap at resolutions of 70,000 and 17,500, separately. Peptides were selected using the high-energy collision dissociation mode with a normalized collision energy setting of 27.0. MS/MS data were obtained using a data-dependent procedure to capture the 15 most abundant precursor ions. An electrospray voltage of 1.6 kV was applied, and the dynamic exclusion duration was set for 15 s.

### 4.7. Protein Identification and Quantification

Raw data files from the Orbitrap were merged and transformed into the MASCOT generic format (MGF) by Proteome Discoverer 1.3 (ThermoFisher Scientific, San Jose, CA, USA). The MS/MS data were searched by three search engines (MyriMatch v2.2.8634, X!Tandem v2015.04.01.1 and MS-GF+v2016.06.29) through IPeak against the Uniprot database of *Ricinus communis* with the following parameters: carbamidomethyl (C), iTRAQ 8 plex (N-term), and iTRAQ 8 plex (K) as fixed modification; oxidation (M) and iTRAQ 8 plex (Y) as variable modifications; full cleavage by trypsin with one missed cleavage permitted; peptide mass tolerance at ±10 ppm and fragment mass tolerance ±0.05 Da [82]. After MS/MS searching, IQuant was used for protein quantification with VSN normalization [83]. The false discovery rate (FDR) at both peptide spectrum matching (PSM) and protein levels was set ≤1%. Proteins with at least one unique peptide were used for quantification. T-tests were used to calculate a *p*-value for each protein. Only ratios with fold change > 1.2 and *p*-value < 0.05 were considered as significant.

### 4.8. Bioinformatics Analysis

Functional annotations of DAPS were conducted using GO (http://www.geneontology.org). The COG (http://www.ncbi.nlm.nih.gov/COG/) database was used for the functional classification of DAPS. The Kyoto Encyclopedia of Genes and Genomes (KEGG) (http://www.genome.jp/kegg/) was used to predict the main metabolic pathways and biochemical signal transduction pathways that the DAPS were involved in. A *p*-value < 0.05 was used as the threshold to determine the significant enrichments of GO and KEGG pathways.

### 4.9. Enzyme-Linked Immunosorbent Assays 

Cold-and control-treated imbibed seeds were ground into powder with liquid nitrogen. 5 g powder was placed in a stoppered flask with 25 mL of 60% aqueous methanol solution and 20 mL of petroleum ether and shaken for 10 min. The homogenate was filtrated by filter paper and the filtrate was collected in the separatory funnel and released in the lower layer (60% aqueous methanol extract) after stratification. The extract was diluted to 30% of the final methanol concentration as the sample to be tested. The content of G6PDH, 6PGL, β-CT subunit of Het-ACCase, BC subunit of Het-ACCase, KASI, KASII, acetyl-CoA, ATP, and UFA were measured using reagent kits from the Jianglai Biotechnology Company Limited of Shanghai, China (Cat.No. JL-F22767, JL-F46429, JL-F46473, JL-F46442, JL-F46461, JL-F46461, JL-F14042, JL13631, JL45670). Briefly, the purified antibody was used to coat microtiter plate wells to make a solid-phase antibody. 50 µL of different standard concentrations (kit available) was added to the coated wells. Blank and testing sample wells were set separately. 40 µL of sample dilution and 10 µL of testing samples were added to the testing sample wells and gently mixed. 100 µL of HRP-conjugate reagents were added to every well except the blank control well, and incubated for 60 min at 37 °C. After incubation, liquid in the well was discarded. The well was washed using a wash solution 5 times and dried. 50 µL chromogen solution A and 50 µL chromogen B were added to each well under dark incubation for 15 min at 37 °C. The reaction was terminated by the addition of a stop solution and color change was measured using a microplate reader at a wavelength of 450 nm.

Sucrose content was determined by an assay kit from Nanjing Jiancheng Bioengineering Institute, Jiangsu, China (A099-1). Briefly, a homogenate of cold-and control-treated imbibed seeds were prepared by the same method as ELISA. The content of sucrose was assayed by measuring the product of sucrose hydrolysis at 290 nm. Data are presented as means of three biological replicates ±SD. Results were considered statistically significant at *p* value < 0.05 using the Student’s *t*-test.

### 4.10. RNA Extraction and qRT-PCR Analysis

Total RNA was extracted from the seeds imbibed at 4 °C and 30 °C for 12 h using a TaKaRa MiniBEST Plant RNA Extraction Kit according to the manufacturer’s instructions (Code No. 9769). RNA integrity were measured using the Agilent 2100 Bioanalyzer (Agilent Technologies, Palo Alto, CA) with RNA integrity number (RIN) >7. Total RNA was treated with DNaseI to remove genomic DNA contamination. cDNA was synthesized, starting with 200 ng of total RNA and using UEIris RT-PCR system for First-Strand cDNA Synthesis (US Everbright, Suzhou, China). qRT-PCR was carried on qTOWER2.2 (Analytik Jena, Jena, Germany) using 2 × SYBR Green qPCR Master Mix (US Everbright, Suzhou, China) according to the manufacturer’s instructions. qRT-PCR was performed with 2 µL of each cDNA diluted at 1:5. Three independent biologically replicated experiments were set up with three technical replicates per experiment. All expression data were normalized against the expression levels of the *RcActin* (XM_002522148) and *RcGAPDH* (XM_002513282) applied as internal standards. The relative levels of eight genes were analyzed using the 2^−ΔΔ*C*t^ method [84]. The primers were designed according to the corresponding nucleotide sequences of castor bean in phytozome v10.2 (http://phytozome.jgj.doe.gov/pz/portal.html). Gene-specific and internal genes primers for qRT-PCR analysis are presented in Table S1. The values were means ±SD. Data were considered statistically significant at *p* value < 0.05 using the Student’s *t*-test.

## 5. Conclusions

Imbibition is a critical process during seed germination. To identify the DAPS that contribute to seed germination under cold stress, the changes of proteomic in 12 h early imbibed seeds under cold stress were investigated in this study. A total of 127 DAPS were identified; these DAPS were involved in carbohydrate and energy metabolism, translation, ribosomal structure and biogenesis, posttranslational modification, protein turnover, chaperones, lipid transport and metabolism, signal transduction etc. These processes can work cooperatively to establish the beneficial equilibrium of physiological and cellular homeostasis. This approach identified new proteins that were not previously known to be associated with cold stress response. Future work will focus on improving cold tolerance via overexpression of β-ketoacyl-acyl carrier protein synthase I and II, and investigating the relationship between accumulation of SnRK1 gamma subunit and seed germination under cold stress. In summary, our proteomics analysis of early imbibed seeds not only increased our understanding of molecular mechanisms associated with castor seed germination under cold stress but also laid the theoretical foundation for the creation of a new cold-tolerant castor bean, and provided a reference for the improvement of cold-tolerant traits in other important economic crops.

## Figures and Tables

**Table 1:** Significantly enriched pathway annotation of DAPS identified by iTRAQ.

No.	Pathway	Number of DAPS	*p*-value
1	Fatty acid biosynthesis	5	0.016388
2	Biotin metabolism	3	0.017208
3	Fatty acid metabolism	6	0.031722
4	Cyanoamino acid metabolism	3	0.033034
5	Ribosome	12	0.044644

**Table 2:** Comparison of expression pattern at the mRNA and protein level of DAPS.

Protein ID	Description	iTRAQ Ratio	*p* Value	qPCR Ratio	*p* Value	Trends ^a^	
						iTRAQ	qPCR
B9S696	dehydrin Xero	1.45 ± 0.133	0.004	4.52 ± 0.009	0.000	+	+
B9RCA6	GPX	1.62 ± 0.136	0.001	2.30 ± 0.116	0.003	+	+
B9RTR0	LEA D-34	1.75 ± 0.138	0.000	5.34 ± 0.512	0.013	+	+
B9SB19	PP2C	1.21 ± 0.114	0.029	6.40 ± 0.952	0.029	+	+
Q41134	KASII	1.38 ± 0.207	0.023	2.76 ± 0.501	0.050	+	+
B9SVJ9	SnRK1 α catalytic subunit	1.32 ± 0.162	0.022	3.85 ± 0.530	0.032	+	+
B9S3Z7	LEA D-34	1.30 ± 0.150	0.021	1.16 ± 0.117	0.232	+	=
B9STQ5	IF5A	1.46 ± 0.279	0.032	0.42 ± 0.073	0.121	+	=

^a^ +: up-regulated; =: not significantly changed.

**Table 3:** Information of DAPS in ribosome pathway.

Protein Accession	Fold Change	Accumulated	Description
B9SKD1	1.25	Up	60S ribosomal protein L3
B9RMF8	1.21	Up	Zn-dependent exopeptidases superfamily protein
B9S4D5	1.24	Up	40S ribosomal protein S26
B9SKG4	1.27	Up	40S ribosomal protein S11
B9SBM0	1.25	Up	60S ribosomal protein L9
B9SIV4	1.35	Up	60S ribosomal protein L7a
B9R982	1.21	Up	40S ribosomal protein S9
B9RG16	1.23	Up	40S ribosomal protein S27
B9SYV4	1.23	Up	60S ribosomal protein L21
B9RQ66	1.43	Up	60S ribosomal protein L28
B9SCT8	1.22	Up	60S ribosomal protein L10a
B9T040	1.5	Up	60S ribosomal protein L35

**Table 4:** Information of DAPS in fatty acid biosynthesis.

Protein Accession	Fold Change	Accumulated	Description
Q41134	1.38	Up	β-ketoacyl-acyl carrier protein synthase II
Q41135	1.45	Up	β-ketoacyl-acyl carrier protein synthase I
B9S1E2	1.26	Up	Biotin caboxylase (BC) subunit of Het-ACCase
B9TAH3	1.47	Up	β-carboxyltransferase (β-CT) subunit of Het-ACCase
B9RF47	1.38	Up	Short chain dehydrogenase

## Supplementary Materials

Supplementary materials can be found at http://www.mdpi.com/1422-0067/20/2/355/s1.

## Abbreviations

iTRAQ	Isobaric tag for relative and absolute quantification
DAPS	Differential abundance protein species
ELISA	Enzyme-linked immunosorbent assays
DEGs	Differentially expressed genes
2-DE	Two-dimensional electrophoresis
GO	Gene ontology
COG	Clusters of Orthologous Groups of proteins
KAS	β-ketoacyl-acyl carrier protein synthase
6PGL	6-phosphogluconolactonase
G6PDH	Glucose-6-phosphate 1-dehydrogenase
GPX	Glutathione peroxidase
LEA	Late embryogenesis abundant protein
SnRK1	SNF1-related protein kniase
IF5A	Eukaryotic translation initiation factor 5A
PP2C	Protein phosphatase 2c
